# Biological motion stimuli are attractive to medaka fish

**DOI:** 10.1007/s10071-013-0687-y

**Published:** 2013-10-20

**Authors:** Tomohiro Nakayasu, Eiji Watanabe

**Affiliations:** Laboratory of Neurophysiology, National Institute for Basic Biology, Higashiyama 5-1, Myodaiji-cho, Okazaki, Aichi 444-8787 Japan

**Keywords:** Biological motion, Motion information, Shoaling behaviour, Medaka (*Oryzias latipes*), Fish

## Abstract

In many social fish species, visual cues play an important role in inducing shoaling behaviour. The present study is the first to examine whether and how “biological motion” depicting a moving creature by means of only a small number of isolated points induces shoaling behaviour in fish. Medaka (*Oryzias latipes*) were used because they are known to have high visual acuity and exhibit a strong tendency to form shoals. In experiment 1, we found that the presentation of medaka biological motion resulted in heightened shoaling behaviour when compared with that of non-biological motion (depicted by a small number of points placed at fixed distances that moved at a constant speed in a constant direction). In experiment 2, it was indicated that medaka biological motion was more effective at inducing shoaling behaviour when compared with human biological motion. In experiment 3, it was demonstrated that shoaling behaviour was largely dependent on the smoothness of the biological motion. In experiment 4, we revealed that shoaling behaviour was maximised in normal speed group and decreased in faster- and slower-than-normal speed groups. In experiment 5, it was shown that shoaling behaviour was slightly reduced when a reversed movie was presented. These results suggest that motion information extracted from conspecifics was sufficient to induce shoaling behaviour in medaka and that deviation from normal and familiar motion impeded shoaling behaviour. The naturalness of motion may be responsible for the induction of shoaling behaviour.

**Electronic supplementary material:**

The online version of this article (doi:10.1007/s10071-013-0687-y) contains supplementary material, which is available to authorized users.

## Introduction

Many teleost fish species form social aggregations. The aggregations of fishes, or shoals, can provide multiple benefits to shoal members. Examples include a reduction in predation risk (Morgan and Godin 1985; Landeau and Terborgh 1986), enhanced feeding opportunities (Pitcher et al. 1982; Morgan and Colgan 1987), and hydrodynamic advantages during locomotion (Svendsen et al. 2003). In addition to the adaptive significance of shoaling, the factors influencing the choice of shoal mate have been well studied. The conventional method to investigate the shoal mate choice is to observe the spontaneous preference between two potential shoal mates using a side-by-side presentation. A subject is separated from shoal mates with a clear glass or plastic bottle, which allowed visual contact but not olfactory and physical contact. It has been reported that visual cues, including shoal size (Lachlan et al. 1998; Pritchard et al. 2001; Ruhl and McRobert 2005; Agrillo et al. 2008), sex (Ruhl and McRobert 2005; Agrillo et al. 2008), familiarity (Lachlan et al. 1998), body colouration (McRobert and Bradner 1998; Engeszer et al. 2004), and body size (Ranta and Lindström 1990; Krause and Godin 1994; Lachlan et al. 1998), affect the shoal mate choice.

Although previous studies have primarily focused on the role of morphological cues (such as body colouration and shape) on shoaling, visual motion cues may also be involved in the induction of shoaling. Pritchard et al. (2001) examined whether the shoal mate choice is influenced by the degree of activity of shoal mates in zebrafish. They manipulated the activity of shoal mates by changing the water temperature (in cold water, the activity was reduced). It was indicated that the subject animals spent more time near the shoals in warm water (25 °C) than near the shoals in cold water (15 °C). These results suggest that zebrafish prefer to shoal with more active individuals. Imada et al. (2010) analysed shoaling behaviour in similar-sized small fish, including medaka, dwarf pufferfish, and zebrafish. Coordinated movement in a pair of medaka was found in homospecific pairs regardless of body colour, sex, or albino mutation, but was not detected between heterospecific pairs (i.e. medaka–pufferfish and medaka–zebrafish pairs). Imada et al. (2010) noted that the coordinated movement may be based on the interaction with a conspecific individual and that the interaction may be mediated by factors such as movement pattern and morphology (but not body colouration or body size). These previous studies suggest that physical motion cues may contribute to the induction of shoaling behaviour. However, real fishes were used as the stimulus animals in these studies. Because researchers have difficulty controlling the behaviour of living animals, using real fishes as stimulus animals would not be appropriate when undertaking a detailed analysis of properties of visual motion, which are critical in the induction of shoaling behaviour. Instead, computer-generated motion stimuli are appropriate.

Computer animation can be a useful and effective tool to study fish behaviour in the laboratory environment because this approach enables us to manipulate single parameters of complex stimulus. Mate choice (Turnell et al. 2003; Robinson-Wolrath 2006; Baldauf et al. 2009; Butkowski et al. 2011), predator evasion (Gerlai et al. 2009; Luca and Gerlai 2012), and shoal preference (Rosenthal and Ryan 2005; Saverino and Gerlai 2008; Abaid et al. 2012; Neri 2012) have been analysed with animated images. It appears, however, that all of these studies depicted the appearance of a real fish. Such social stimuli include colour and shape information, which are sufficient to affect fish behaviours. To conduct the detailed analysis of visual motion, which is involved in the induction of shoaling behaviour, we will need to extract the motion cues from living animals and remove other physical characteristics.

To date, it is largely unknown how fish species detect and process the motion of conspecific fish. However, a number of studies in humans have investigated the processing of other individuals’ motion pattern. Johansson (1973) found that when presented with an animation sequence consisting of a small number of dots strategically placed on the major joints of the human body, observers immediately interpreted the movement pattern of isolated points as a human figure. He termed such stimuli “biological motion”. The use of biological motion stimuli has an advantage because it allows us to isolate and present motion information. Since Johansson’s pioneering work, the perceptual cues of biological motion and the neural mechanisms mediating the perception of biological motion have been investigated extensively in humans (for reviews, see Giese and Poggio 2003; Troje 2008). In recent years, the ability to perceive biological motion has been investigated in non-human animals, including chimpanzees (Tomonaga 2001), baboons (Parron et al. 2007), rhesus monkeys (Oram and Perrett 1994; Vangeneugden et al. 2010; Jastorff et al. 2012), common marmosets (Brown et al. 2010), bottlenosed dolphins (Herman et al. 1990), cats (Blake 1993), rats (MacKinnon et al. 2010; Foley et al. 2012), pigeons (Omori 1997; Dittrich et al. 1998; Troje and Aust 2013), and chicks (Regolin et al. 2000; Vallortigara et al. 2005; Vallortigara and Regolin 2006; Miura and Matsushima 2012). Unfortunately, it has not been studied whether fish can perceive biological motion and what effects biological motion has on fish behaviours.

To reveal whether and how physical movement cues contribute to the induction of shoaling, five experiments were conducted using biological motion stimuli. Medaka were used for this study because they are known to have a high visual acuity (Carvalho et al. 2002; Beck et al. 2004; Tsubokawa et al. 2009; Matsunaga and Watanabe 2010, 2012) and exhibit a strong tendency to form shoals (Nakamura 1952; Imada et al. 2010). All stimuli were presented to the subject medaka on a cathode ray tube (CRT) display. In the present study, shoaling behaviour was assessed through the analysis of the time during which the medaka were close to the display and their travel distance in the test tank. Experiment 1 compared the effects of biological motion and non-biological motion stimuli on shoaling behaviour. Biological motion stimuli depicting a moving creature by means of only a small number of isolated dots were generated based on the analyses of free-moving medaka. Non-biological motion stimuli were depicted by a small number of dots placed at fixed distances that moved at a constant speed in a constant direction. In experiment 2, medaka and human biological motion stimuli were compared. Although human biological motion involved more complex movements than non-biological motion, both the form (the configuration of dots) and motion information of human biological motion were changed from medaka biological motion. In experiment 3, we degraded the motion information without affecting the form information. We degraded the motion information by using displays in which the same frame was repeatedly presented while maintaining the average speed of the moving dots. This allowed us to examine how such jerky types of biological motion influence shoaling behaviour. Experiment 4 examined the effects of changes in the speed of biological motion. There were five swimming speeds: normal speed, two faster-than-normal speeds, and two slower-than-normal speeds. We investigated whether movement speed modulates shoaling behaviour. In experiment 5, we manipulated the temporal order of biological motion. The effects of forward and reverse playback movies on shoaling behaviour were compared.

## Materials and methods

### Animals and housing conditions

Adult medaka (*Oryzias latipes*, black variety) were used. In experiments 1 and 3, medaka were purchased from a pet shop, Medaka Honpo (Hiroshima, Japan), and in experiments 2, 4, and 5, Focus (Kumamoto, Japan). They were maintained in 23-L glass aquaria for at least 7 days prior to the start of the experiment. The stock populations (approximately 40 fish per aquarium) were kept in aerated and filtered water at 26 ± 1 °C. The holding water was prepared by mixing deionised water and artificial sea salt (6.9 g/23 L; Tetra Marine Salt Pro; Tetra Japan, Tokyo, Japan). The lighting cycle was 12-h light and 12-h dark (light from 08:00 to 20:00). The animals were fed an artificial dry diet (Tetra Killifish Food; Tetra Japan, Tokyo, Japan) twice a day (at 09:00 and 17:00). After the study, they were transferred to retirement aquaria and maintained. All experiments were approved by the committee for Animal Experimentation at the National Institutes of Natural Sciences, Japan (approval number: 12A018 and 13A036).

### Motion tracking of medaka

**Fig. 1:**
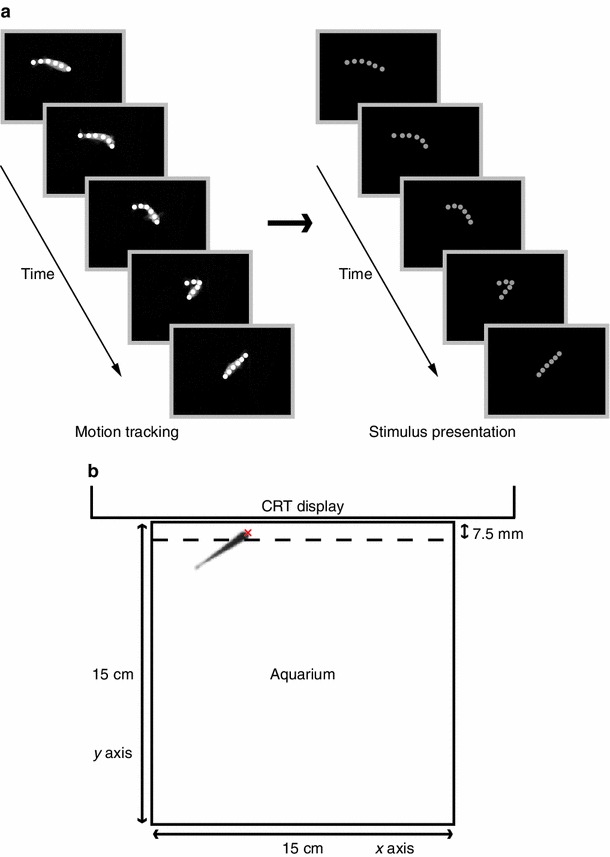
A schematic representation of the method. **a** An animation sequence depicting biological motion. Six points were automatically placed along the body trunk of a moving fish using computer software. Based on the tracking data, the movements of medaka were expressed as those of six *grey dots*. **b** A schematic of the experimental set-up for the behavioural test. Stimuli were presented on a CRT display. The tip of the head of the fish was automatically tracked using computer software. We analysed the time that the head was near the CRT display (an area of 7.5 mm in width from the inner surface of the tank on the display side), the travel distance on the *x* axis (in a *horizontal* direction against the display), and the travel distance on the *y* axis (in a *vertical* direction against the display)

To create biological motion stimuli, motion tracking was conducted by using medaka purchased from Medaka Honpo. A cubic aquarium (inner side length of 15 cm) was used. The aquarium was filled with housing water. The water depth was 8.0 cm. The temperature of the tank water was maintained at 26 ± 1 °C by air conditioning. The bottom and three sides of the tank, excluding the side where the video camera was positioned, were covered with matte-black plastic material to prevent a reflection of the illumination. Illumination at the surface of the water was adjusted to 7,000 lx using four white florescent lamps that were placed near the tank. In most of the animal biological motion studies, it appears that the biological motion stimulus was constructed based on the video recording of only one animal. However, if we choose an unusual individual, exceptional behavioural patterns may be incidentally recorded. To overcome such problems, we used multiple individuals. Additionally, in zebrafish, it was indicated that while males were preferred less by other males, both sexes were attracted to females (Ruhl and McRobert 2005). Therefore, four females (Medaka 1–4) were used in the present study. One minute of motion tracking was conducted from 11:00 to 15:00. Each medaka was transferred to the test tank 30 min before motion tracking began. The movements of medaka were recorded from the side and above of the tank using digital video cameras (Himawari GE60; Library, Tokyo, Japan). The video images (640 × 480 pixels) were recorded at 60 frames per second (fps) and analysed using motion analyser software (Wriggle Tracker; Library, Tokyo, Japan). In previous animal studies, the recorded video images have been transformed into biological motion patterns by manual tracking of the positions of the major joints (e.g. Vallortigara et al. 2005; Jastorff et al. 2012). However, such methods cannot be applied to fish, which do not have visible joints. Therefore, in the present study, a small number of points were automatically placed at equal distance along the body trunk in each video frame using Wriggle Tracker (Fig. 1a). Six points were used.

### Stimulus presentation

All animation sequences were presented on a 15-in CRT display with a refresh rate of 60 Hz and resolution of 1,024 × 768 pixels. Visual stimuli were controlled by Psychlops software [C++ library for developing psychophysical stimulus; please refer to our previous study, Watanabe et al. (2010)] running on a Windows PC. Because of the dimensional limits of a computer display, biological motion stimuli of medaka were constructed based solely on the data of two-dimensional coordinates from the side view. Biological motion stimuli were expressed as the movements of six grey dots (28 cd/m^2^, Psychlops oval function 3 pixels in diameter; Fig. 1a; Online Resource 1) on a black background and presented within an area of 577 × 308 pixels (150 × 80 mm^2^; interpixel distance 0.26 mm) located in the centre area of the display. As noted above, motion tracking to generate biological motion stimuli was conducted for 1 min, which corresponded to 3,600 frames. Each frame was presented sequentially at a frame rate of 60 fps. The duration of one cycle was 1 min. Four biological motion stimuli were generated based on the tracking data of four individuals (Medaka 1–4).

### Behavioural test

Cubic glass aquaria (inner side length of 15 cm) were used as test tanks for the behavioural test. To restrict the exterior visual stimulation, the lateral sides were covered with white polystyrene sheets. The test tanks were filled with housing water (water depth 8.0 cm). The tank water was maintained at a temperature of 26 ± 1 °C by air conditioning.

The behavioural test consisted of a habituation period (23 h + 1 h), baseline period (1 min), and stimulus presentation period (4 min). A naïve fish was randomly selected from the stock populations and individually transferred to the test tank (from 09:00 to 17:00). Twenty-three hours later, the test tank was moved to the test room and attached to the CRT display, and the animal was allowed to habituate for 1 h. The illumination at the surface of the water was adjusted to 2,500 lx with two white florescent lamps. After the habituation period, the behaviour of the animal was continuously recorded for 5 min. The first 1 min was the baseline period, in which no stimuli were presented. Visual stimuli were presented on the CRT display during the last 4 min (stimulus presentation period). The subjects were presented with 4-min biological motion animations, which constituted four repetitions of the same 1-min animation. The videos were recorded at a frame rate of 30 fps. To alleviate the effort of behavioural analysis, many frames were deleted from the original sequence using Super Bara-Baby X software (LNSOFT, Fukuoka, Japan). The frame rate was reduced to 3 fps (a reduction in frame rate had little effect on the outcome of the statistical analysis).

Behavioural analysis was conducted using DIPP-Motion 2D software (DITECT, Tokyo, Japan). The coordinates of the tip of the fish’s head were automatically tracked in each video frame (Fig. 1b). The amount of time that the head was in close range to the CRT display (an area of 7.5 mm in width from the inner surface of the tank on the display side) was calculated. In addition, we measured the travel distance of the head (*x* axis, the horizontal direction against the display; *y* axis, the vertical direction against the display). Shoaling behaviour was assessed based on the time spent near the display and the travel distance on the *x* axis. However, we did not use the simple mean value because subjects which incidentally tend to spend long time near the display are regarded as having higher shoaling tendencies. Then, we excluded the medaka that stayed close to the display (within 7.5 mm) for an excessive amount of time (over 56 s) during the baseline period, and calculated the differences in behavioural measures between the baseline and the stimulus presentation periods. The difference values were statistically analysed.

### Experiment 1: Comparison of biological and non-biological motion stimuli

In experiment 1, we compared the effects of biological and non-biological motion stimuli on shoaling behaviour. The visual stimuli were constructed from the tracking data of Medaka 1–4. The mean distance between the centre of points and the mean movement speed of the centre of the mass, respectively, were as follows: Medaka 1, 5.07 mm and 77.46 mm/s; Medaka 2, 5.24 mm and 70.62 mm/s; Medaka 3, 5.28 mm and 49.17 mm/s; and Medaka 4, 5.31 mm and 45.39 mm/s. Four non-biological motion stimuli were produced based on these mean values. Six grey dots placed at fixed distances (5.07, 5.24, 5.28, or 5.31 mm, respectively) moved at a constant speed (77.46, 70.62, 49.17, or 45.39 mm/s, respectively) in a constant direction (Online Resource 2). As with biological motion, non-biological motion stimuli were presented at a frame rate of 60 fps. The starting coordinate of the head dot of non-biological motion was the centre of the display, and the movement direction was randomly determined. If non-biological motion stimuli exited the presentation area (577 × 308 pixels, located on the centre of the display), they bounced off in random directions. Thus, there were a number of differences between these two visual stimuli. In non-biological motion, the six dots always formed a straight line, and the distance between adjacent dots was fixed (i.e. the configuration of dots was always shaped as the letter “I”). However, in biological motion, the relative position of the dots and the distance between the dots changed constantly. Occasionally, the six dots were shaped as letters “C”, “J”, and “L”, and they overlapped considerably. The movement patterns of non-biological motion were also distinct from those of biological motion. The dots in non-biological motion did not accelerate, decelerate, or hover. The dots in non-biological motion only turned in some other direction when they exited the presentation area, but the dots in biological motion frequently changed their moving direction within the area. Furthermore, while the dots around the tail fin in biological motion moved more than did those of other body parts, the travel distance of the six dots in non-biological motion were all identical.

As noted above, the subjects were presented with biological motion animations, which constituted four repetitions of the same 1-min animation. For non-biological motion, the position of the head dot was reset to the centre of the display every 1 min. Based on the behavioural analysis of the baseline period, six medaka were excluded, leaving 64 subjects. Half of the subjects were presented with biological motion stimuli (BM group, *n* = 32), and the other half were presented with non-biological motion stimuli (NBM group, *n* = 32). In each group, half of the subjects were male, and the other half were female. In the BM group, one-quarter of the subjects, i.e. eight subjects, were exposed to one of four biological motion stimuli (based on the tracking data of Medaka 1–4). Similarly, 8 of the 32 subjects in the NBM group were exposed to one of four non-biological motion stimuli. Because of the small sample size, the data from the male and female subjects and from the four variations in stimuli were pooled. In all the following experiments, half of the subjects were male, and the other half were female. In addition, one-quarter of the subjects were presented with one of four visual stimuli based on the data from Medaka 1–4.

### Experiment 2: Comparison of medaka and human biological motion

Experiment 2 investigated the effects of presentation of biological motion stimuli derived from other species. Non-biological motion stimuli in experiment 1 did not depict the complexity of movement of creatures. A lack of complexity of motion may have critical effects on the induction of shoaling behaviour. We therefore presented medaka with visual stimuli with complex motion, such as biological motion stimuli derived from other species. In the present study, we used human biological motion stimuli.

Biological motion stimuli of medaka were presented as in experiment 1 (M group). Human biological motion animations were generated using a modified version of Cutting’s algorithm (Cutting 1978), and we presented medaka with human biological motion (H group). There were six dots corresponding to the head, shoulder, wrists, and ankles. The walker faced left. The size of the walker was determined based on the tracking data of Medaka 1–4. The mean distances between the centre of the head dot and the centre of the ankle dots were as follows: 25.35, 26.2, 26.4, or 26.55 mm. A gait cycle was completed in 1,500 ms, which consisted of 90 animation frames, resulting in a walking speed of 40 cycles per minute. Human biological motion moved at a constant speed (77.46, 70.62, 49.17, or 45.39 mm/s, respectively) in a constant direction as with experiment 1 (Online Resource 3). The starting coordinate of the centre of the mass of human biological motion was the centre of the display, and the movement direction was randomly determined. Based on the time spent in proximity to the display during the baseline period, four medaka were removed. Subjects were assigned to either M or H groups (each *n* = 32).

### Experiment 3: Effects of degradation of motion information

In experiment 3, we examined the effects of degradation of motion information by using jerky types of biological motion displays (derived from medaka) in which the same frame was repeatedly presented while maintaining the average speed of the dots. If motion information is involved in the induction of shoaling behaviour, the degradation of motion information would reduce the effect of biological motion on shoaling behaviour.

Biological motion stimuli in experiment 1 were presented at the rate of 60 fps, i.e. each frame was presented only once (1, 2, 3, 4, 5, 6, 7, 8, 9…; the numbers indicate the frames of the original sequence). In addition to the smoothly moving biological motion (60FPS group), we created jerky types of biological motion by manipulating the number of times each frame was presented while maintaining the moving speed as follows (Online Resource 4): 15FPS group (1, 1, 1, 1, 5, 5, 5, 5, 9…); 10FPS group (1, 1, 1, 1, 1, 1, 7, 7, 7…); 5FPS group (1, 1, 1…1, 1, 1, 13, 13, 13…); and 1FPS group (1, 1, 1…1, 1, 1, 61, 61, 61…). In the 15FPS, 10FPS, 5FPS, and 1FPS groups, the same frames were presented 4, 6, 12, and 60 times, respectively. Eight medaka that remained close to the display for a long time during the baseline period were excluded from the results, resulting in 120 subjects. An equal number of subjects were assigned to each of five groups: 60FPS, 15FPS, 10FPS, 5FPS, or 1FPS (each *n* = 24).

### Experiment 4: Effects of speed manipulation

In experiments 4 and 5, we examined what characteristics of motion are involved in the induction of shoaling behaviour. We focused our attention on the movement speed (experiment 4) and temporal order (experiment 5). There has been considerable research on the effects of speed modulation on the processing of biological motion in humans (e.g. Kozlowski and Cutting 1977; Barclay et al. 1978; Lange and Lappe 2006; Watanabe 2008; Cai et al. 2011). Barclay et al. (1978) examined how variations in walking speed (normal or slow speed) affected the recognition of gender. Human participants could identify the gender of biological motion walkers when the motion sequence was presented at a normal speed but were unable to identify the gender of slow-moving biological motion walkers. In agreement with the results of Barclay et al. (1978), Kozlowski and Cutting (1977) indicated that the female walkers were unable to be identified as female when the speed was slower than normal. However, they also found that an increase in walking speed was associated with an increase in correct identification (in addition, for the male walkers, the correct identification could be made regardless of speed). In humans, slow-moving biological motion may be difficult to interpret, but an increase in the speed may have little impact on the interpretation of biological motion or even improve the performance (see also Lange and Lappe 2006).

From the original tracking coordinate data, we calculated the coordinates of hypothetical frames between the original frames (e.g. the coordinates of frame 1.5 were intermediate between the coordinates of frames 1 and 2). In the normal speed group, the original frames were presented at a rate of 60 fps (1× group: 1, 2, 3, 4, 5, 6…; the numbers indicate the frames of the original sequence). Biological motion stimuli were also presented as follows (Online Resource 5): double speed (2× group: 1, 3, 5, 7, 9, 11…); one-and-a-half speed (1.5× group: 1, 2.5, 4, 5.5, 7, 8.5…); half speed (0.5× group: 1, 1.5, 2, 2.5, 3, 3.5…); and quarter speed (0.25× group: 1, 1.25, 1.5, 1.75, 2, 2.25…). The original recorded duration of each biological motion was 1 min. Because of the manipulation of the speed, the durations of one stimulus sequence for the 2×, 1.5×, 0.5×, and 0.25× groups were 30, 40 s, 2, and 4 min, respectively. To equate the total stimulus duration, the stimulus sequence was repeated 4, 8, 6, 2, and 1 times for the 1×, 2×, 1.5×, 0.5×, and 0.25× groups, respectively. Therefore, the total duration of the stimulus presentation was 4 min for each group. Four medaka were excluded from the results based on the time spent in proximity to the display during the baseline period, resulting in 120 subjects. An equal number of subjects were assigned to each of the five groups: 1×, 2×, 1.5×, 0.5×, and 0.25× (each *n* = 24).

### Experiment 5: Effects of reverse playback

In experiment 5, we investigated whether medaka are sensitive to changes in biological motion elicited by reverse playback. In experiments 1–4, the original medaka biological motion stimuli (e.g. biological motion in experiment 1) and manipulated stimuli (e.g. non-biological motion in experiment 1) consisted of different sets of video frames. Therefore, the stimulus differences introduced by the manipulations in experiments 1–4 were much large. However, the sets of video frames were identical between the forward and reverse movies, although the frames were displayed in the opposite order between the two stimuli. The reverse playback of the movie was highly selective manipulation of the motion, and this experiment would be quite informative to enhance our understanding of how biological motion stimuli are processed by medaka. Neri (2012) examined the effects of reverse playback of social visual stimuli on shoaling behaviour in zebrafish. He used the animated images in which the appearance of a real fish was depicted (such stimuli included the shape and stripe pattern information). Zebrafish displayed spontaneous preference for the forward movie over the reverse movie. It appears that the discrimination between the forward and reverse movies is not so difficult in case where social stimuli contain the rich amount of information. However, other previous studies have suggested that it is difficult to accurately interpret the reverse playback of impoverished displays. Pavlova et al. (2002) examined how a movie of biological motion shown in reverse was processed by humans. They found that a reverse movie was not interpreted as the reversed version of a forward movie. Vangeneugden et al. (2010) also indicated that three rhesus monkeys needed a very long training period (18,023, 37,238, and 43,576 trials, respectively) to distinguish forward and reverse walking using impoverished displays in which the joints were connected by cylinder-like primitives (thus, these displays were slightly richer in information than biological motion stimuli).

The stimulus sequence was presented in two manners: forward playback (F group: 1, 2, 3, 4, 5, 6…; the numbers indicate the frames of the original sequence) and reverse playback (R group: 3,600, 3,599, 3,598, 3,597, 3,596, 3,595…; Online Resource 6). Both stimuli (the forward and reverse movies) consisted of exactly the same set of frames. Playing the frames in reverse order left the form information (the configuration of dots) unaltered. However, this manipulation changed some information about the motion. Accelerating motions became decelerating ones and vice versa. Furthermore, the head dots in the reversed movie were identical to the ones around tail fin in the forward movie. Thus, in the reversed movie, the head dots moved more than did those of other body parts. Based on the time spent near the display during the baseline period, three medaka were excluded from the results. Subjects were assigned to either F or R groups (each *n* = 32).

### Statistical analysis

The mean total length of the fish was analysed using a one-way analysis of variance (ANOVA). Behavioural measures in the baseline period were also assessed via a one-way ANOVA. Behavioural changes from the baseline period were assessed using a two-way ANOVA with time as a within-subject factor (baseline 1, 2, 3, and 4 min) and group as a between-subject factor (BM and NBM groups in experiment 1; M and H groups in experiment 2; 60FPS, 15FPS, 10FPS, 5FPS, and 1FPS groups in experiment 3; 1×, 2×, 1.5×, 0.5×, and 0.25× groups in experiment 4; F and R groups in experiment 5). If the interaction was found to be significant, the simple main effects were analysed. When necessary, Ryan’s method was used for post hoc multiple comparisons. A probability level of *p* < 0.05 was adopted as the level of statistical significance. All data are expressed as the mean ± SEM.

## Results and discussion

### Experiment 1

The mean total length of the medaka did not differ between the BM and NBM groups (31.57 ± 0.29 mm and 31.46 ± 0.28 mm, respectively; *F*(1,62) = 0.07, *p* > 0.05). In the baseline period, the time during which the fish were close to the display (12.30 ± 2.66 s and 15.47 ± 2.89 s, respectively; *F*(1,62) = 0.63, *p* > 0.05), the travel distance on the *x* axis (in the horizontal direction against the display; 727.82 ± 70.67 mm and 851.98 ± 102.17 mm, respectively; *F*(1,62) = 0.97, *p* > 0.05), and the travel distance on the *y* axis (in the vertical direction against the display; 1,124.77 ± 158.21 mm and 1,236.03 ± 173.24 mm, respectively; *F*(1,62) = 0.22, *p* > 0.05) did not differ between the BM and NBM groups. The total length of the fish and the behavioural measures during the baseline period were equivalent between groups.

**Fig. 2:**
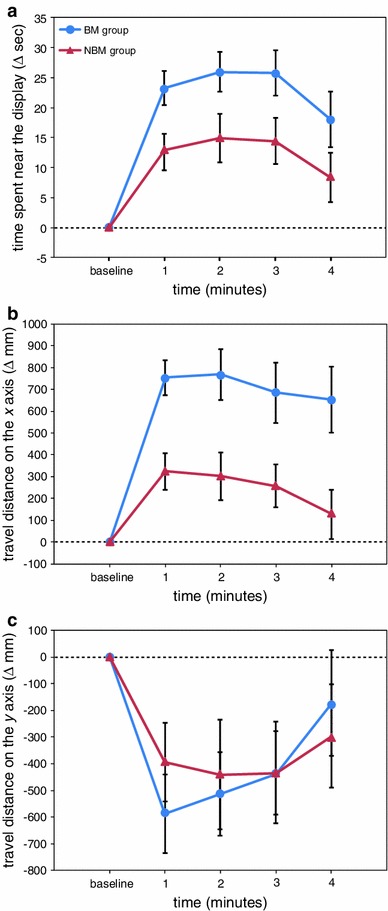
The results of experiment 1, in which the effects of biological motion (BM group) were compared with those of non-biological motion (NBM group). **a** The time during which medaka were close to the display, **b** the travel distance on the *x* axis, and **c** the travel distance on the *y* axis

The effects of the stimulus presentation are represented in Fig. 2. With regard to the change in the time spent in proximity to the display (Fig. 2a), the two-way ANOVA revealed significant main effects of group (*F*(1,62) = 5.59, *p* < 0.05) and time (*F*(4,248) = 22.27, *p* < 0.001). The BM group spent significantly more time near the display than the NBM group, and exposure to visual stimuli significantly increased the time spent near the display.

Regarding the change in the travel distance on the *x* axis (Fig. 2b), the two-way ANOVA revealed significant main effects of group (*F*(1,62) = 11.14, *p* < 0.01) and time (*F*(4,248) = 17.82, *p* < 0.001) and a significant interaction effect between group and time (*F*(4,248) = 3.91, *p* < 0.01). The increase in the travel distance on the *x* axis was significantly higher in the BM group than the NBM group over the entire stimulus presentation period (*p* < 0.05).

With respect to the change in the travel distance on the *y* axis (Fig. 2c), there was a significant main effect of time (*F*(4,248) = 8.68, *p* < 0.001), but group differences were not detected (*p* > 0.05).

The medaka were shown to spend more time near the display when presented with biological motion compared with non-biological motion (Fig. 2a). Furthermore, the medaka presented with images consisting of biological motion patterns moved more horizontally against the display than the medaka presented with non-biological motion patterns (Fig. 2b), although the travel distance in the vertical direction was largely reduced in both groups (Fig. 2c). These results indicate that biological motion stimuli have a large effect on the induction of shoaling behaviour and are highly attractive to medaka. As with human newborns (Simion et al. 2008) and infants (Fox and McDaniel 1982), common marmosets (Brown et al. 2010), and chicks (Vallortigara et al. 2005; Vallortigara and Regolin 2006; Miura and Matsushima 2012), medaka attended to biological motion patterns to a great extent.

### Experiment 2

The total length of the medaka and the behavioural indices during the baseline period did not differ between M and H groups (data not shown, all *p* > 0.05).

**Fig. 3:**
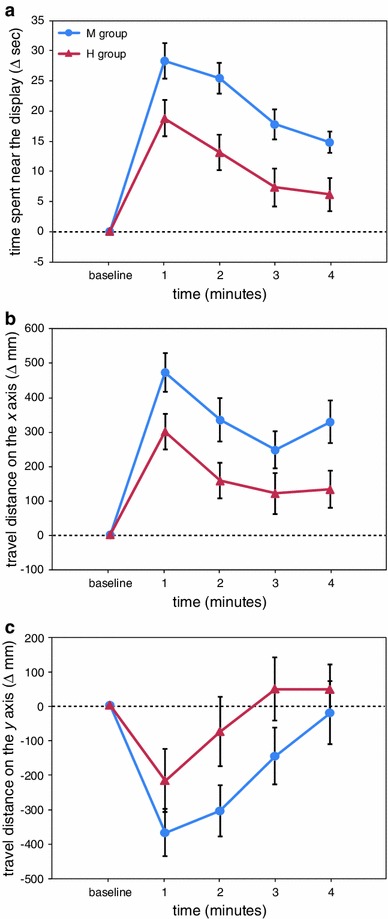
The results of experiment 2, in which the effects of medaka biological motion (M group) were compared with those of human biological motion (H group). **a** The time during which medaka were close to the display, **b** the travel distance on the *x* axis, and **c** the travel distance on the *y* axis

Figure 3 depicts the effects of the stimulus presentation. As for the change in the time spent near the display (Fig. 3a), the two-way ANOVA revealed significant main effects of group (*F*(1,62) = 10.13, *p* < 0.01) and time (*F*(4,248) = 45.93, *p* < 0.001) and a significant interaction effect between group and time (*F*(4,248) = 3.24, *p* < 0.05). The increase in the time spent near the display was significantly higher in the M group than the H group over the entire stimulus presentation period (*p* < 0.05).

Figure 3b shows the change in the travel distance on the *x* axis. The two-way ANOVA indicated that significant main effects of group (*F*(1,62) = 6.35, *p* < 0.05) and time (*F*(4,248) = 25.10, *p* < 0.001). The travel distance on the *x* axis in the M group was significantly higher than the H group, and the presentation of the visual stimuli significantly increased the travel distance on the *x* axis.

For the change in the travel distance on the *y* axis (Fig. 3c), there was a significant main effect of time (*F*(4,248) = 12.57, *p* < 0.001), but we found no significant group differences (*p* > 0.05).

Although human biological motion depicted the complexity of movement of creatures, it was not as effective as medaka biological motion at inducing increases in both the time spent near the display (Fig. 3a) and the travel distance in *x* axis (Fig. 3b). The present results indicate that visual stimuli with complex motion were not necessarily attractive to medaka and that medaka were highly sensitive to biological motion derived from conspecifics.

As with the BM group (in experiment 1), the presentation of medaka biological motion (M group) significantly increased the time spent near the display (Fig. 3a) and the travel distance on the *x* axis (Fig. 3b) compared with the baseline period. The supplier of medaka in this experiment was different from that in experiment 1. The change in suppliers had little influence on the performance during the behavioural test; thus, it appears that the phenomenon that biological motion can stimulate shoaling behaviour is robust and reliable.

### Experiment 3

As with experiments 1 and 2, the total length of the medaka and the behavioural indices during the baseline period did not differ between the five groups (data not shown, all *p* > 0.05).

**Fig. 4:**
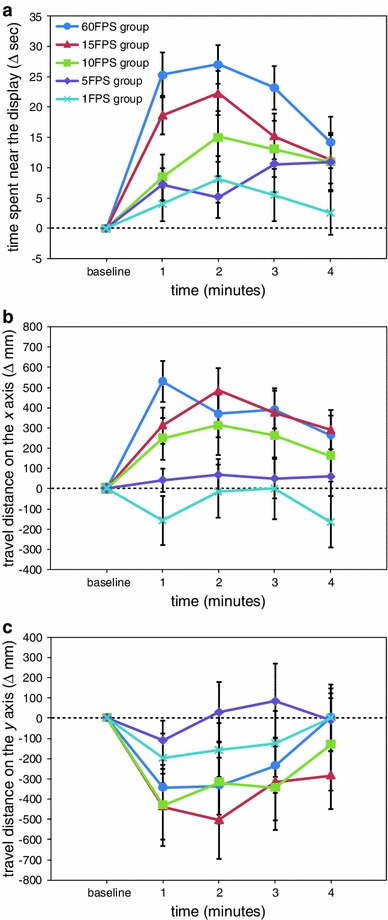
The results of experiment 3, in which the effects of the degradation of motion information were examined. In addition to the smoothly moving biological motion (60FPS group), jerky types of biological motion were presented (15FPS, 10FPS, 5FPS, and 1FPS groups). **a** The time during which medaka were close to the display, **b** the travel distance on the *x* axis, and **c** the travel distance on the *y* axis

Figure 4 represents the effects of the stimulus presentation. For the change in the time spent near the display (Fig. 4a), the two-way ANOVA indicated significant main effects of group (*F*(4,115) = 5.01, *p* < 0.001) and time (*F*(4,460) = 22.82, *p* < 0.001) and a significant interaction effect between group and time (*F*(16,460) = 2.30, *p* < 0.01). While visual stimuli were presented, the time spent in proximity to the display in the 60FPS and 15FPS groups remained significantly increased from baseline (*p* < 0.05). In the 10FPS group, the time spent near the display at the 1-min time point was comparable with that of the baseline period, but thereafter, it remained significantly prolonged (*p* < 0.05). However, in the 5FPS and 1FPS groups, the visual stimulus presentation did not significantly affect the time spent near the display (*p* > 0.05). As a result, at the 1–3-min time points, significant group differences were found as follows (all *p* < 0.05): at the 1-min time point, the 60FPS group versus the 10FPS, 5FPS, and 1FPS groups, and the 15FPS group versus the 1FPS group; at the 2-min time point, the 60FPS and 15FPS groups versus the 5FPS and 1FPS groups; and at the 3-min time point, the 60FPS group versus the 1FPS group.

Figure 4b shows the change in the travel distance on the *x* axis. The two-way ANOVA indicated significant main effects of group (*F*(4,115) = 5.08, *p* < 0.001) and time (*F*(4,460) = 7.57, *p* < 0.001) and a significant interaction effect between group and time (*F*(16,460) = 1.83, *p* < 0.05). Over the whole period of stimulus presentation, the travel distance on the *x* axis in the 60FPS and 15FPS groups was significantly increased compared with the baseline period (*p* < 0.05). However, in the other three groups, exposure to the visual stimuli did not significantly influence the travel distance on the *x* axis (*p* > 0.05). As a result, significant group differences were found as follows (all *p* < 0.05): at the 1-min time point, the 60FPS, 15FPS, and 10FPS groups versus the 1FPS group, and the 60FPS group versus the 5FPS group; at the 2-min time point, the 15FPS group versus the 5FPS and 1FPS groups; and at the 4-min time point, the 60FPS and 15FPS groups versus the 1FPS group.

As for the change in the travel distance on the *y* axis (Fig. 4c), there was a significant main effect of time (*F*(4,460) = 6.34, *p* < 0.001), but we found no significant group differences (*p* > 0.05).

The degradation of motion information by repeated presentation of the same frame had critical effects on the induction of shoaling behaviour. The presentation of smoothly moving biological motion (60FPS group) resulted in significant increases in both the time spent near the display (Fig. 4a) and the travel distance on the *x* axis (Fig. 4b). Similar to the 60FPS group, the 15FPS group, in which the smoothness of the animation was slightly reduced, showed great sensitivity to biological motion (Fig. 4a, b). Although the 10FPS group somewhat attended to the biological motion animation, the 5FPS and 1FPS groups (in which jerkier types of biological motion were presented) showed little response to the animation (Fig. 4a, b). These data suggest that shoaling behaviour was highly dependent on the smoothness of the biological motion.

### Experiment 4

The total length of the medaka and the behavioural indices during the baseline period did not differ between the five groups (data not shown, all *p* > 0.05).

**Fig. 5:**
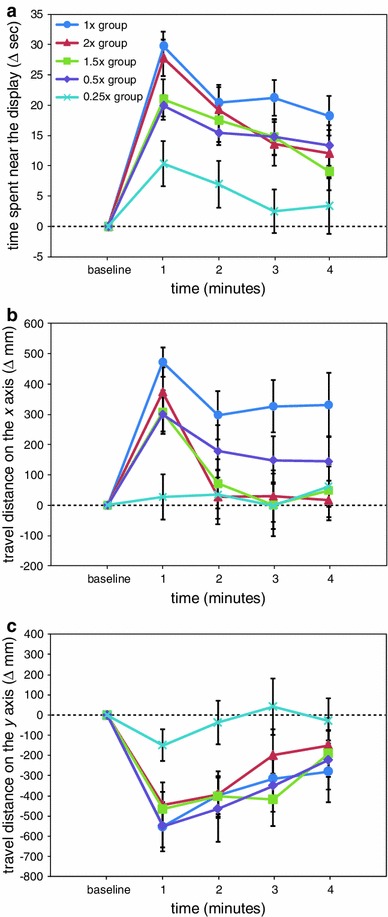
The results of experiment 4, in which the speed of biological motion was manipulated. Biological motion was presented at the normal speed (1× group), the two faster-than-normal speeds (2× and 1.5× groups), and the two slower-than-normal speeds (0.5× and 0.25× groups). **a** The time during which medaka were close to the display, **b** the travel distance on the *x* axis, and **c** the travel distance on the *y* axis

The effects of the presentation of visual stimuli are depicted in Fig. 5. Figure 5a shows the change in the time during which the medaka were close to the display. The two-way ANOVA revealed significant main effects of group (*F*(4,115) = 5.29, *p* < 0.001) and time (*F*(4,460) = 56.83, *p* < 0.001) and a significant interaction effect between group and time (*F*(16,460) = 1.95, *p* < 0.05). Over the whole period of the stimulus presentation, the time spent in proximity to the display in the 1×, 2×, 1.5×, and 0.5× groups was significantly increased compared with the baseline period (*p* < 0.05). In the 0.25× group, the time spent near the display was significantly prolonged at the 1-min time point (*p* < 0.05) but returned to baseline thereafter. As a result, significant group differences were found as follows (all *p* < 0.05): at the 1- and 2-min time points, the 1× and 2× groups versus the 0.25× group; at the 3-min time point, the 1×, 2×, 1.5×, and 0.5× groups versus the 0.25× group; and at the 4-min time point, the 1× group versus the 0.25× group.

With respect to the change in the travel distance on the *x* axis (Fig. 5b), the two-way ANOVA indicated significant main effects of group (*F*(4,115) = 3.32, *p* < 0.05) and time (*F*(4,460) = 18.42, *p* < 0.001) and a significant interaction effect between group and time (*F*(16,460) = 2.19, *p* < 0.01). While the visual stimuli were presented, the travel distance on the *x* axis in the 1× group remained significantly increased from baseline (*p* < 0.05). In the 0.5× group, a significant increase in the travel distance on the *x* axis was found at the 1-min time point (*p* < 0.05), and the increase was attenuated thereafter. The travel distance on the *x* axis in the 2× and 1.5× groups was significantly increased transiently at the 1-min time point compared with the baseline period (*p* < 0.05) but returned to baseline thereafter. In the 0.25× group, the visual stimulus presentation did not significantly influence the travel distance on the *x* axis (*p* > 0.05). As a result, significant group differences were found as follows (all *p* < 0.05): at the 1-min time point, the 1×, 2×, 1.5×, and 0.5× groups versus the 0.25× group; at the 3-min time point, the 1× group versus the 2×, 1.5×, and 0.25× groups; and at the 4-min time point, the 1× group versus the 2× group.

Regarding the change in the travel distance on the *y* axis (Fig. 5c), the two-way ANOVA showed a significant main effect of time (*F*(4,460) = 21.82, *p* < 0.001). However, group differences were not detected (*p* > 0.05).

Changes in speed were found to have profound effects on the induction of shoaling behaviour. The increase in the time spent near the display in the two faster-than-normal speed groups (2× and 1.5× groups) was not significantly different from that of the 1× group (Fig. 5a). However, although the travel distance on the *x* axis in the 1× group remained significantly increased while the visual stimuli were presented, the travel distance on the *x* axis in the 2× and 1.5× groups significantly increased immediately after the presentation of the visual stimuli but returned to baseline thereafter (Fig. 5b). We infer that the fast-moving biological motion stimuli were easily detectable but that such types of biological motion were too fast to follow and/or swim parallel to. Both the time spent in proximity to the display (Fig. 5a) and the travel distance on the *x* axis (Fig. 5b) were increased in the 0.5× group, in which mildly slow-moving biological motion was presented, although not as much as in the 1× group. However, the presentation of very slow-moving biological motion (0.25× group) had little impact on both indices of shoaling behaviour.

Because of the speed manipulation, not only the speed but also the motion cycles were changed. In the two faster-than-normal speed groups, the duration of one sequence was shorter, and the number of repetitions was higher compared with the normal speed group. In the two slower-than-normal speed groups, the opposite was true. Shoaling behaviour was reduced in both faster- and slower-than-normal speed groups compared with the normal speed group. The present results suggest that deviation from the normal speed impeded shoaling behaviour. It thus seems that motion speed, not the duration of one stimulus sequence or the number of sequence repetitions, is the critical determinant of the induction of shoaling behaviour. Although Watanabe (2008) and Cai et al. (2011) also suggested that the duration of one stimulus sequence and the number of sequence repetitions did not play an important role in the processing of biological motion (see also Neri et al. 1998), we must address this issue in future work in which a variety of factors are systematically manipulated.

### Experiment 5

As with the above four experiments, the total length of the medaka and the behavioural measures during the baseline period did not differ between the F and R groups (data not shown, all *p* > 0.05).

**Fig. 6:**
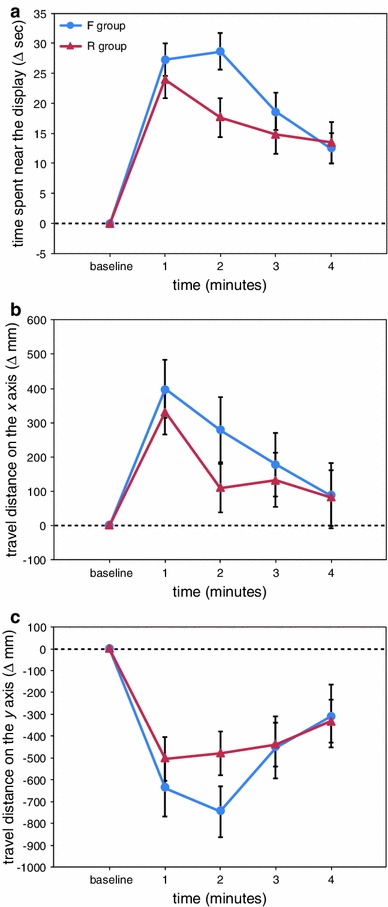
The results of experiment 5, in which the effects of the forward playback movie (F group) were compared with those of the reverse playback movie (R group). **a** The time during which medaka were close to the display, **b** the travel distance on the *x* axis, and **c** the travel distance on the *y* axis

Figure 6 shows the effects of the stimulus presentation. Regarding the change in the time spent near the display (Fig. 6a), the two-way ANOVA showed a significant main effect of time (*F*(4,248) = 49.65, *p* < 0.001) and a significant interaction effect between group and time (*F*(4,248) = 2.70, *p* < 0.05). This interaction was because the time spent in proximity to the display was significantly longer in the F group compared with the R group at the 2-min time point (*p* < 0.05).

Regarding the change in the travel distance on the *x* axis (Fig. 6b), the two-way ANOVA showed a significant main effect of time (*F*(4,248) = 13.85, *p* < 0.001) but found no group differences (*p* > 0.05).

As for the change in the travel distance on the *y* axis (Fig. 6c), there was a significant main effect of time (*F*(4,248) = 27.46, *p* < 0.001), but group differences were not detected (*p* > 0.05).

Experiment 5 examined the effects of reverse playback on the shoaling behaviour. The medaka in the F group were presented with biological motion stimuli identical to those used in experiments 1–4. We replicated the above findings, in which shoaling behaviour was induced by biological motion stimuli. While the stimulus differences between groups in experiments 1–4 were large, the reverse playback of the movie could selectively manipulate the visual stimuli. Therefore, the analysis of the effects of reverse playback was of value. However, unfortunately, the forward movie was slightly more effective at inducing shoaling behaviour than the reverse movie. The present results suggest that changes in the temporal order of biological motion caused only a small reduction in shoaling behaviour. Using impoverished displays, it is likely that detecting the differences between the forward and reverse playback movies was difficult. Vangeneugden et al. (2010) showed that in rhesus monkeys, discrimination between forward and reverse movies using impoverished displays (in which the body features were represented by cylinder-like primitives) was considerably difficult. Perhaps the temporal order information of biological motion (i.e. the cue that distinguishes between the forward and reverse playbacks of biological motion animations) may have low perceptual saliency for animals.

## General discussion

Using biological motion stimuli, which depict a moving creature by means of just a small number of isolated points, the present study examined the importance of physical motion information in the induction of shoaling behaviour. Shoaling behaviour was assessed based on the time during which medaka were close to the display on which the visual stimuli were presented and their travel distance in the test tank. The results of experiment 1 showed that medaka presented with biological motion spent a long time in proximity to the display and moved more horizontally against the display. In experiment 2, we found that the presentation of biological motion derived from conspecifics (medaka) induced higher increases in the time spent near the display and horizontal movement against the display when compared with the presentation of biological motion derived from other species (humans). In experiment 3, it was shown that the time during which medaka were close to the display and the travel distance in the horizontal direction against the display were largely dependent on the smoothness of biological motion; jerky types of biological motion did not stimulate shoaling behaviour. Experiment 4 demonstrated that shoaling behaviour was substantially influenced by speed manipulation of biological motion. When presented with fast-moving biological motion, medaka spent a long time near the display but did not have as much horizontal movement against the display; medaka showed little reaction to very slow-moving biological motion. The results of experiment 5 indicated that there were minor differences between the effects of forward and reverse playbacks of biological motion (the time spent near the display was slightly reduced in medaka presented with the reverse movie). In all experiments, we found that deviation from familiar and normal motion inhibited the induction of shoaling behaviour. It appears that the naturalness of motion contributed to the induction of shoaling behaviour.

The processing of motion cues could play a key role in survival. The ability to detect and interpret the movement of other living organisms would be of importance in the recognition of conspecifics, protection against predators, and the success of mating or hunting. It has indeed been shown that many animal species are very sensitive to physical motion information by using biological motion stimuli. However, previous biological motion studies were conducted only in mammalian and avian species. For example, Blake (1993) successfully trained cats to discriminate a biological motion pattern of a walking cat from patterns without biological motion. Vallortigara et al. (2005) reported that newly hatched chicks exhibit a spontaneous preference to approach stimuli depicting biological motion over non-biological motion (rigid motion and random motion). The present study indicated that medaka fish attended to biological motion stimuli. It is likely that the visual mechanisms for the detection of biological motion stimuli are evolutionarily more conserved than previously thought.

Many fish species prefer to shoal with individuals of similar appearance to themselves. McRobert and Bradner (1998) examined the effects of body colouration on shoal choices in black and white mollies. When given a choice between a group of black mollies and a group of white mollies, black and white mollies each spent more time near the group of mollies similar to their own colouration. Ranta and Lindström (1990) investigated whether body size is an important cue for inducing shoaling behaviour in three-spined sticklebacks. Small sticklebacks were found to associate with the group of small conspecific fish, and large sticklebacks preferred the shoal of large-sized conspecific fish. Although it appears that several factors, such as hunger (Reebs and Saulnier 1997) and early experience (Engeszer et al. 2004), have an impact on the preference for individuals of similar appearance, many fish species have generally been found to choose shoal mates that are phenotypically similar to themselves.

The present study examined the contribution of motion information to the induction of shoaling behaviour using biological motion stimuli, in which a living creature was described with only a small number of dots, removing morphological characteristics. The present study demonstrated that the motion information of conspecifics was sufficient to elicit shoaling behaviour. It was shown that while changes in the temporal order had only a slight reduction effect on shoaling behaviour, speed manipulation had a great impact on the induction of shoaling behaviour. The indices of shoaling behaviour (the time spent in proximity to the display and travel distance in the horizontal direction against the display) were the highest in the normal speed group and decreased in the faster- and slower-than-normal speed groups. In humans, it appears that the alternation in normal movement speed leads to odd perceptions; in extreme cases, a fast-moving biological motion walker is viewed as a fast and mechanical, robot-like figure, and a slow-moving walker appears to be on the moon (see Barclay et al. 1978). The same holds true for medaka; to human eyes, fast-moving biological motion was similar to a pinball, and slow-moving biological motion looked as if it were in a viscous liquid. It is likely that a low familiarity of motion was associated with the inhibition of shoaling behaviour. Previous studies have shown that several fish species prefer to shoal with familiar individuals (e.g. Griffiths and Magurran 1997; Lachlan et al. 1998; Edenbrow and Croft 2012). For example, Lachlan et al. (1998) presented guppies with a choice between familiar and unfamiliar shoal mates (subjects and familiar shoal mates had been housed in groups for 14 days prior to testing) and revealed that 12 of 15 subjects preferentially chose familiar mates. Fish may shoal with familiar individuals due to their adaptive benefits, such as an increase in cooperative anti-predator behaviours (Chivers et al. 1995), a reduction in aggressive behaviours (Utne-Palm and Hart 2000), and the facilitation of the social learning of foraging behaviour (Swaney et al. 2001). It is likely that deviation from familiar (and natural) movement speed caused the reduction in shoaling behaviour.

However, although both fast- and slow-moving visual stimuli would be unfamiliar to medaka, speed manipulation differentially affected the induction of shoaling behaviour, depending on whether the speed was increased or decreased. While subjects spent a significant amount of time near the display when fast-moving biological motion was presented (though they did not have much horizontal motion against the display), the presentation of very slow-moving biological motion hardly elicited any behavioural responses. In human studies, an increase in the speed of biological motion had little impact on (or even improved) the performance of gender discrimination and of walking direction discrimination, but a decrease in the speed interfered with the interpretation of biological motion (Kozlowski and Cutting 1977; Barclay et al. 1978; Cai et al. 2011). The present findings are consistent with the results of earlier studies with humans, although it remains to be determined why the manipulation of biological motion speed had differential effects on the performance, depending on whether the speed was increased or decreased. To address this issue, we should systematically manipulate a number of factors, such as the duration of one stimulus sequence and the number of sequence repetitions, although Watanabe (2008) and Cai et al. (2011) suggested that these factors were not critical in humans.

The present study is the first to use biological motion stimuli in a fish species. There are still some issues that were not addressed in the present study. The first issue is why differential changes in speed have differential effects on shoaling behaviour, as discussed above. The second issue is whether the induction of shoaling behaviour by the presentation of biological motion is based on an innate disposition or learning over the course of development. The question that the detection of biological motion is an intrinsic capacity of the visual system or is an experience-dependent phenomenon has so far been examined only in humans (Fox and McDaniel 1982; Simion et al. 2008) and chicks (Vallortigara et al. 2005). In humans, while Fox and McDaniel (1982) reported that a preference for biological motion patterns started from about 4 to 6 months of age, Simion et al. (2008) showed that 2-day-old newborns preferentially attended to biological motion displays. Newly hatched chicks, reared and hatched in darkness, exhibited a preference for biological motion patterns over non-biological motion patterns (Vallortigara et al. 2005). Although some previous studies have suggested that the detection of biological motion is an innate capacity of the visual system, this issue was not addressed in medaka. The third issue is the validity of indices of shoaling behaviour. In previous studies, shoaling behaviour has been quantified by analysing the approach tendency towards conspecifics (e.g. Ruhl and McRobert 2005; Tobler and Schlupp 2008). The present study also assessed shoaling behaviour on the basis of the two behavioural indices (the time spent in proximity to the display and horizontal movement against the display), which represent the approach tendency towards visual motion. In experiment 2, it was indicated that the increases in both indices were higher for medaka presented with visual stimuli derived from conspecifics than for medaka presented with visual motion derived from humans. It is likely that the changes in the behavioural measures for assessing shoaling behaviour were not because medaka merely reacted to visual stimuli with complex motion, but because medaka responded sensitively to visual stimuli derived from conspecifics. However, we depicted a moving fish by means of only a small number of isolated points. Because the biological motion stimuli are substantially different from real medaka, the fact that medaka have a high tendency to approach the biological motion stimuli does not guarantee that they recognised the movements of a small number of points as a moving medaka. Further analysis on the behavioural patterns elicited by the presentation of biological motion stimuli would contribute to find a more valid measure of shoaling behaviour. The fourth issue is the ability of the fish to process more detailed information from biological motion. It is known that humans can perceive a variety of information from biological motion stimuli, including agents (e.g. a human, dog, or bird) of motion (Mather and West 1993; Pavlova et al. 2001), actions (Johansson 1973; Dittrich 1993), gender (Kozlowski and Cutting 1977; Barclay et al. 1978), the identity of familiar individuals (Cutting and Kozlowski 1977), emotion (Dittrich et al. 1996), and the weight of an object being lifted (Runeson and Frykholm 1981). In the future, we should create various types of biological motion and assess whether medaka are sensitive to the manipulation of stimulus properties. Such studies would shed light on the mechanisms for processing biological motion in fish species.

In the present study, we demonstrated that biological motion stimuli were highly attractive to medaka and that the naturalness of motion was critical in the induction of the behavioural responses to biological motion. However, further studies are needed to reveal the mechanisms underlying the visual processing of biological motion. Morphological characteristics and motion (or behavioural) characteristics can be valuable in recognising animal species, sex, and group members. Studies using biological motion stimuli will enhance our understanding of how non-human animals extract and process the information which is vital for their survival.

## Electronic supplementary material

Below is the link to the electronic supplementary material.

Online Resource 1. A sample of the biological motion animation. The luminance and diameter of the dots in this demonstration were changed from those in the original to enhance the visibility of stimulus. A CRT display was used in the actual experiments. However, to create this demonstration, the biological motion was displayed on a liquid crystal display (LCD) and recorded. Therefore, a residual image has been considerably detected because of the slow response speed of the LCD. The text (“Biological motion”) and scale bar in the movie did not appear in the actual stimulus (MPG 1854 kb)

Online Resource 2. A sample of the non-biological motion (MPG 1854 kb)

Online Resource 3. A sample of human biological motion (MPG 1854 kb)

Online Resource 4. A sample of jerky types of biological motion: the upper left is presented to the 15FPS group; the upper right is presented to the 10FPS group; the lower left is presented to the 5FPS group; and the lower right is presented to the 1FPS group (MPG 2014 kb)

Online Resource 5. A sample of fast- and slow-moving biological motion: the upper left is presented to the 2× group; the upper right is presented to the 1.5× group; the lower left is presented to the 0.5× group; and the lower right is presented to the 0.25× group (MPG 2014 kb)

Online Resource 6. A sample of the reverse playback movie (MPG 1854 kb)
